# Moderate alcohol consumption as risk factor for adverse brain outcomes and cognitive decline: longitudinal cohort study

**DOI:** 10.1136/bmj.j2353

**Published:** 2017-06-06

**Authors:** Anya Topiwala, Charlotte L Allan, Vyara Valkanova, Enikő Zsoldos, Nicola Filippini, Claire Sexton, Abda Mahmood, Peggy Fooks, Archana Singh-Manoux, Clare E Mackay, Mika Kivimäki, Klaus P Ebmeier

**Affiliations:** 1 Department of Psychiatry, University of Oxford, Warneford Hospital, Oxford OX3 7JX, UK; 2 FMRIB Centre, Nuffield Department of Clinical Neurosciences, University of Oxford, Oxford, OX3 9DU, UK; 3 University of Oxford, Warneford Hospital, Oxford, OX3 9DU, UK; 4 Department of Epidemiology and Public Health, University College London, London, WC1E 6BT, UK

## Abstract

**Objectives** To investigate whether moderate alcohol consumption has a favourable or adverse association or no association with brain structure and function.

**Design** Observational cohort study with weekly alcohol intake and cognitive performance measured repeatedly over 30 years (1985-2015). Multimodal magnetic resonance imaging (MRI) was performed at study endpoint (2012-15).

**Setting** Community dwelling adults enrolled in the Whitehall II cohort based in the UK (the Whitehall II imaging substudy).

**Participants** 550 men and women with mean age 43.0 (SD 5.4) at study baseline, none were “alcohol dependent” according to the CAGE screening questionnaire, and all safe to undergo MRI of the brain at follow-up. Twenty three were excluded because of incomplete or poor quality imaging data or gross structural abnormality (such as a brain cyst) or incomplete alcohol use, sociodemographic, health, or cognitive data.

**Main outcome measures** Structural brain measures included hippocampal atrophy, grey matter density, and white matter microstructure. Functional measures included cognitive decline over the study and cross sectional cognitive performance at the time of scanning.

**Results** Higher alcohol consumption over the 30 year follow-up was associated with increased odds of hippocampal atrophy in a dose dependent fashion. While those consuming over 30 units a week were at the highest risk compared with abstainers (odds ratio 5.8, 95% confidence interval 1.8 to 18.6; P≤0.001), even those drinking moderately (14-21 units/week) had three times the odds of right sided hippocampal atrophy (3.4, 1.4 to 8.1; P=0.007). There was no protective effect of light drinking (1-<7 units/week) over abstinence. Higher alcohol use was also associated with differences in corpus callosum microstructure and faster decline in lexical fluency. No association was found with cross sectional cognitive performance or longitudinal changes in semantic fluency or word recall.

**Conclusions** Alcohol consumption, even at moderate levels, is associated with adverse brain outcomes including hippocampal atrophy. These results support the recent reduction in alcohol guidance in the UK and question the current limits recommended in the US.

## Introduction

Alcohol use is widespread and increasing across the developed world.^1^ ^2^ ^3^ It has historically been viewed as harmless in moderation,^4^ defined variably from 9-18 units (72-144 g) a week.^5^ ^6^ Recent evidence of associations with risk of cancer^7^ has prompted revision of UK government alcohol guidance, though US Federal Dietary guidelines (2015-20) allow up to 24.5 units a week for men.^8^ Even light drinking (midpoint <12.5g daily/8 units a week) has been associated with increased risk of oropharnygeal, oesophageal, and breast cancer.^7^ ^9^ While chronic dependent drinking is associated with Korsakoff syndrome and alcoholic dementia,^10^ the long term effects of non-dependent alcohol consumption on the brain are poorly understood. Robust evidence of adverse associations would have vital implications for public health.

Some authors have suggested an inverted U shaped relation between alcohol use and brain outcomes, similar to that seen with cardiovascular disease. Light-to-moderate drinking has been associated with a lower risk of dementia^11^ ^12^ and a reduced incidence of myocardial infarction^13^ and stroke.^14^ Brain imaging studies, however, have thus far failed to provide a convincing neural correlate that could underpin any protective effect. Results of research into the effects of moderate alcohol on the brain are inconsistent.^15^ Moderate alcohol consumption in older people has been associated with reduced total brain volume,^16^ increased ventricle size,^17^ grey matter atrophy,^18^ and reduced density of frontal and parietal grey matter,^19^ ^20^ but others have not found such associations^15^ or only at higher consumptions.^21^ Associations between moderate alcohol consumption and white matter findings are also inconsistent. De Bruin and colleagues reported increased white matter volume in moderate drinkers compared with abstainers,^22^ whereas Anstey and colleagues found the inverse relation.^23^ Similarly, whereas increased white matter hyperintensities have been described in moderate drinkers compared with abstainers,^24^ others found no association.^17^ ^23^ ^25^

Unresolved questions persist because of design limits to existing studies of non-dependent drinking and brain imaging. Alcohol consumption cannot be randomised over long periods. Most studies to date have been cross sectional or with limited prospectively gathered data on alcohol. People typically underestimate their alcohol intake,^26^ a problem likely to be worse in a retrospective study. Studies have also included elderly people, in whom sub-threshold presymptomatic cognitive impairment might already have an impact on drinking patterns.

We used data on alcohol consumption gathered prospectively over 30 years to investigate associations with brain structural and functional outcomes in 550 non-alcohol dependent participants. Our hypotheses were twofold: light drinking (<7 units weekly) is protective against adverse brain outcomes and cognitive decline and heavier drinking (above recommended guidelines) is associated with adverse brain and cognitive outcomes.

## Methods

### Study design and participants

Five hundred and fifty people were randomly selected for the current Whitehall II imaging substudy (2012-15) from the Whitehall II cohort study.^27^ The Whitehall II study was established in 1985 at University College London, with the aim of investigating the relation between socioeconomic status, stress, and cardiovascular health. It recruited 10 308 non-industrial civil servants across a range of employment grades. Sociodemographic, health, and lifestyle variables (including alcohol use) were measured over a follow-up period of about 30 years, at about five year intervals (phase 1: 1985-88, phase 3: 1991-93, phase 5: 1997-99, phase 7: 2003-04, phase 9: 2007-09, phase 11: 2011-12). To make the sample as representative as possible of the cohort at baseline, we drew a random list of 1380 participants from those who took part in the Whitehall II phase 11 clinical examination or phase 10 pilot examination and had consented. Participants were sampled from high, intermediate, and low socioeconomic groups.

Alcohol variables collected in each phase included units drunk a week, frequency of drinking a week over the previous year, and results of the CAGE screening questionnaire.^28^ We used weekly consumption in this analysis as there is less likelihood of a ceiling effect in comparison with drinking frequency. We calculated average alcohol use across the study as mean consumption a week averaged across all study phases. Participants were deemed “abstinent” if they consumed less than 1 unit of alcohol a week. “Light” drinking was defined as between 1 and <7 units a week and “moderate” drinking as 7 to <14 units a week for women and 7 to <21 units for men, based on use in the existing literature and government guidelines (fig 1). “Unsafe drinking” was defined according to pre-2016 (21 units (168 g) a week for men and 14 units (112 g) for women) and newly revised UK Department of Health guidelines (>14 units (112 g) for men and women) and further categorised (14-20, 21-30, >30 units weekly) for the purposes of the logistic regression analysis.^29^ Non-dependent drinkers were defined as those scoring <2 on the CAGE questionnaire.

**Figure f1:**
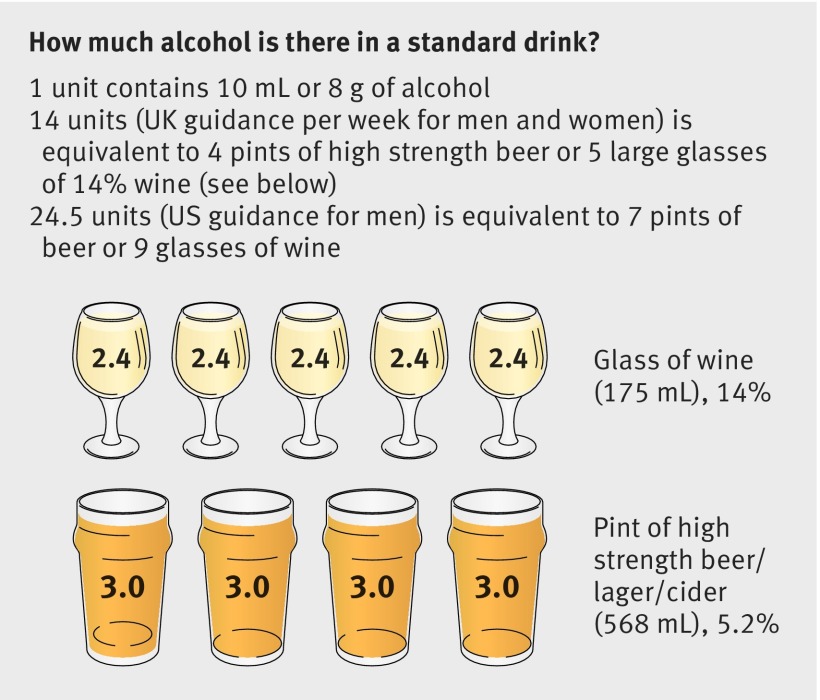
**Fig 1** UK 2016 guidelines on alcohol consumption (see www.alcoholconcern.org.uk/help-and-advice/help-and-advice-with-your-drinking/unit-calculator/) (redrawn from Alcohol Concern, 2016)

Age, sex, education, smoking, social activity—such as attendance at clubs and visits with family/friends, physical activity, voluntary work—and component measures of the Framingham stroke risk score—such as blood pressure, smoking, history of cardiovascular events, cardiovascular drugs—were assessed by self report questionnaire. Social class was determined according to occupation at phase 3 (highest class=1, lowest=4). Drugs (number of psychotropic drugs reported as taken) and lifetime history of major depressive disorder (assessed by structured clinical interview for DSM IV) were assessed at the time of the scan. Information about personality traits was determined by questionnaire at phase 1 and included trait impulsivity (question: “Are you hot-headed?”).

Cognitive function was assessed longitudinally at phases 3, 5, 7, 9, and 11 and at the time of scanning with lexical (how many words beginning with a specific letter can be generated in one minute) and semantic (how many words in a specific category can be named in one minute) fluency tests. Short term memory recall (20 words) was tested at phases 3, 5, 7, 9, and 11. Cross sectional cognitive performance was measured at the time of the scan with the Montreal cognitive assessment (MoCA, education adjusted), trail making test (TMT-A and B), Rey-Osterrieth complex figure (RCF) test (copy, immediate, delay, recognition), Hopkins verbal learning test (HVLT-R; immediate, delay), Boston naming test (BNT), and digit span and digit substitution test (DSST). Full scale IQ (FSIQ) was estimated at the time of the scan with the test of premorbid functioning-UK version (TOPF-UK), with adjustment for sex and education.

Participants were included in the imaging substudy if they were safe to undergo MRI and able to give informed consent. Exclusions were due to incomplete or poor quality imaging data or gross structural abnormality (such as a brain cyst), incomplete data on alcohol use (>2 study phases data missing), and missing sociodemographic, health, or cognitive data (fig 2).

**Figure f2:**
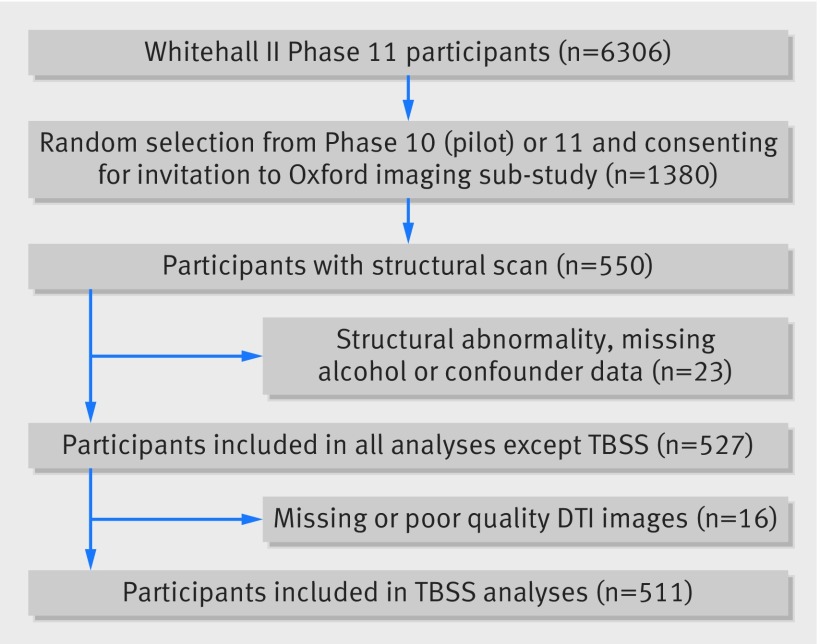
**Fig 2** Flow chart of participants included in analysis alcohol consumption and brain function

### MRI analysis

All MRI scans were acquired at the functional magnetic resonance imaging of the brain (FMRIB) centre, University of Oxford, with a 3 Tesla Siemens Verio scanner (2012-15). We used T1-weighted and diffusion tensor (DTI) 3T MRI sequences for these analyses.^30^

Full technical details are in the appendix. In brief, we initially examined associations between alcohol use and grey matter using voxel based morphometry, an objective method to compare grey matter density between individuals in each voxel (smallest distinguishable image volume) of the structural image. For each participant for subsequent analyses we additionally extracted hippocampal volumes (adjusted for total intracranial volume) using an automated segmentation/registration tool. Automated segmentation of the amygdala was less reliable in this sample so we did not use extracted volumes in this analysis. Three clinicians independently defined hippocampal atrophy according to visual rating (Scheltens score^31^) and reached a consensus.

Diffusion tensor images indicate the directional preference of water diffusion in neural tissue and allow inferences about the structural integrity of white matter tracts. In healthy myelinated fibres diffusion is restricted perpendicular to the longitudinal axis of the fibre—that is, it is anisotropic. We carried out voxel-wise statistical analysis of diffusion tensor data (fractional anisotropy (FA), axial diffusivity (AD), radial diffusivity (RD), and mean diffusivity (MD)) using tract based spatial statistics (TBSS).^32^

### Outcomes

Primary outcomes were continuous measures of grey matter density in the voxel based morphometry analysis and white matter integrity in the tract based spatial statistics analysis (fractional anisotropy, mean, radial, and axial diffusivity).

Visual ratings of hippocampal atrophy were dichotomised into atrophy versus no atrophy based on 0/1 on the (4 point) Scheltens scale to reflect clinical use (“abnormal” versus “normal”).^31^ Hippocampal volume (%intracranial volume) was used as a continuous variable in a multiple linear regression analysis.

As cognitive outcomes we used decline in short term memory, semantic and lexical fluency, and cross sectional performance on Montreal cognitive assessment, trail making test, Rey-Osterrieth complex figure test, Hopkins verbal learning test, Boston naming test, digit span, and digit substitution test.

### Statistical analysis

All analyses were done with R,^33^ unless otherwise stated. To assess representativeness of included participants we examined differences between included and excluded participants using *t* tests of means (continuous variables) or χ^2^ tests of independence (categorical variables). According to variable type, we used means (standard deviations), medians (interquartile ranges), or numbers (percentages) to summarise sociodemographic and clinical measures for included participants who were split by safe versus unsafe average alcohol use averaged over all phases, on the basis of UK contemporary (pre-2016) guidelines. Significant differences between safe and unsafe drinkers in continuous variables were tested with *t* tests of means (normally distributed) or Wilcoxon rank sum tests (non-normally distributed), and in binary categorical variables (and mini-mental state examination, Montreal cognitive assessment, and Framingham stroke risk score, which have lower and upper bounds) with Fisher’s exact test of proportions. In view of small group numbers (<5) for social class, we performed a simulation test to estimate group differences.^34^ Weekly consumption of alcohol (units and grams) was described with means, standard deviations, medians, and interquartile ranges.

We examined alcohol trends over time using mixed effects modelling, with time from study baseline (phase 1) as the independent variable and alcohol consumption (units/week) as the dependent variable. This method accounts for missing data and correlation of repeated measures (in this case alcohol use). We calculated intercepts (baseline consumption) and slopes (trends over study) for each participant. The ability of other variables to predict longitudinal trends of alcohol consumption was tested by inclusion of the following in the mixed effects model: age, sex, education, premorbid IQ, social class, Framingham risk score (a composite measure including smoking, cardiovascular disease or diabetes, cardiovascular drugs), exercise frequency, club attendance, voluntary work, visits with friends and family, lifetime history of major depressive disorder on the structured clinical interview for DSM IV (SCID) (yes-2/no-1), and current psychotropic drugs (yes-2/no-1).

We included mean alcohol consumption (units/week) across all study phases as an independent variable in voxel based morphometry (grey matter density as dependent variable) and tract based spatial statistics analyses (FA/MD/RD/AD as dependent variable). Voxel-wise, we applied a generalised linear model (GLM) using permutation based non-parametric testing (randomise),^35^ correcting for multiple comparisons across space (threshold-free cluster enhancement, TFCE).

We used two post hoc tests to confirm the associations between alcohol consumption and hippocampal size after the voxel based morphometry analysis. Firstly, we used logistic regression to calculate odds ratios for left and right hippocampal atrophy versus no atrophy (visual atrophy ratings based on a cut off of 0/1 on the Scheltens scale),^31^ given average alcohol consumption across study phases. The latter was categorised as abstinent (<1 unit, reference group), 1 to <7 units, 14 to <21 units, 21 to <30 units, and >30 units a week. Secondly, we performed multiple linear regression with hippocampal volume (extracted from FIRST (an automated segmentation/registration tool), adjusted for intracranial volume and transformed by squaring to normalise the residuals) as the dependent variable and alcohol consumption as an independent variable.

In all analyses with a brain measure as the dependent variable, we included the following potential confounding variables (identified from knowledge of the literature) as independent variables: age, sex, premorbid IQ, education, social class, Framingham risk score, current psychotropic drugs (number), lifetime history of major depressive disorder (structured clinical interview: yes-2/no-1), exercise frequency, club attendance, voluntary work, and visits with friends and family. In the subset with data on personality traits (n=179), analyses were additionally adjusted for impulsiveness.

We used mixed effects models to model longitudinal cognitive data. For count data (word recall from list of 20: “memory”) we used a binomial regression and for lexical and semantic fluency (performed within a certain time) we used Poisson regression. The following fixed effects were included: time from study baseline, average alcohol consumption across the study (abstinent (reference group, <1 unit weekly), 1- <7, 7- <14, 14- <21, >21), age, sex, education, social class, premorbid IQ, and Framingham stroke risk score. To test whether cognitive decline significantly differed between abstainers and those with higher alcohol intakes, we added interaction terms between time and alcohol category. Contrasts between other categories of drinking were also checked to test for significant differences in cognitive decline—for example, those drinking 1-<7 versus >21 units. We used Wald tests,^36^ ^37^ estimating the overall effect of all interactions between alcohol and time on the models, to test the null hypothesis that rates of cognitive decline did not differ between alcohol categories. Learning effects have been well demonstrated when the same cognitive test is presented more than once to a participant, which in our study could obscure true cognitive decline. In an attempt to control for this we added a dummy variable to code for the first time the test was taken (First). We also dummy coded for the test being performed at Oxford (Oxford), as there was an atypically short time interval between phase 11 and the last measurement point, which we hypothesised could result in an increased learning effect. We included interaction terms for FSIQ*First and FSIQ*Oxford to check if learning effects differ with premorbid IQ. Participant identification was included as a random effect. Usual diagnostic checks were performed on the models. The resulting coefficients from binomial regression equate to log(odds) and from Poisson regression to log(Poisson mean count). Exponentiated estimates are reported in the appendix. Regression coefficients were converted into interpretable differences in lexical decline per year compared with abstainers by: 100*(1−(exp (estimates)). Models were visually presented with graphs to predict trends in cognitive test scores over the study for a “typical” participant: male, mean age 70, 15 years’ education, social class I, IQ 118, and Framingham stroke risk score 10%.

We fitted regression models to check whether average alcohol consumption over the study (independent variable) predicted cross sectional performance on a range of memory tests (dependent variable) performed at the study end point. Age, sex, education, and premorbid IQ were included as covariates. When the test score represented a continuous variable, we used multiple linear regression. For count data (such as digit coding), we initially fitted Poisson regression and checked for over-dispersion. If this was found, we used a negative binomial model. For the remainder of the tests, where the upper score is bounded, we initially fitted regression models using binomial distributions. If over-dispersion was in evidence we performed a folded transformation and checked for approximate normality using Q-Q plots of residuals. The same models were re-fitted with and without alcohol consumption, and a hypothesis test (likelihood ratio) was performed. Calculated P values were used to test whether alcohol made a significant difference to the model.

Structural equation modelling (SEM; Amos 24 for Windows) was used post hoc for hypothesis testing and to generate fit statistics for models of relations between alcohol use, brain measures, and cognitive decline. This modelling allows simultaneous analysis of multiple variables in one model, and time series with auto-correlated errors. The hypothesised underlying structure of the model was constructed following the voxel based morphometry, tract based spatial statistics, and mixed effects analyses, with average alcohol consumption as an exogenous variable, hippocampal volume, corpus callosum mean diffusivity (generally the most sensitive measure of loss of white matter integrity), and decline in lexical fluency (slopes from mixed effects model) included as endogenous variables (with latent variables to account for measurement error). We modelled covariance of alcohol with sex and IQ and between brain measures. The model was improved by iteratively eliminating paths with P>0.1 and monitoring of the successive improvement of the model fits statistics (χ^2^, comparative fit index, root mean square error of approximation, and the Tucker-Lewis index) until we identified the most parsimonious model.

In all analyses, results were judged significant if the adjusted P value was <0.05. Bootstrapping was performed to derive 95% confidence intervals for estimates.

### Patient involvement

Participants were from the Whitehall II cohort. No patients were involved in setting the research question or the outcome measures, nor were they involved in the design, recruitment, or conduct of the study. No patients were asked to advise on interpretation or writing up of results. Results were disseminated to the study participants in abstract format and as presentations at the 30th anniversary day for the Whitehall II cohort.

## Results

### Participants/descriptive data

Sociodemographic, health, and lifestyle data are reported for the 527 included participants, separated into alcohol consumption groups (table 1). Twenty three participants were excluded from the voxel based morphometry and visual ratings analyses on the basis of structural brain abnormalities, poor quality images, or missing confounder data (fig 2). A further 16 were excluded from the tract based spatial statistics analysis because of missing or poor quality diffusion tensor images. Excluded participants did not significantly differ from those included on any of the reported characteristics (data available on request). There was a higher proportion of men, and participants were slightly less educated, with higher blood pressure and lower measures of depressive symptoms compared with the larger Whitehall II cohort (see appendix table A). Mean age was 43.0 (SD 5.4) at the start of the study (appendix table B). Unsafe drinkers differed from safe drinkers by having a higher premorbid IQ, a higher percentage of men and smokers, and higher Framingham risk scores (table 1).

**Table 1 tbl1:** Baseline (phase 1 unless otherwise indicated) summary characteristics of 527 participants (unless marked) included in analysis by safe (<14 units/week for women, <21 units/week for men) and unsafe alcohol consumption, defined by contemporaneous (pre-2016) UK Department of Health guidelines, on average over study duration

	Safe drinkers (n=428)	Unsafe drinkers (n=99)	Difference between groups or other statistic (95% CI)
Mean (SD) age at start (years)	43.0 (5.4)	42.8 (5.1)	MD=0.2 (−1.0 to 1.4), P=0.7
No (%) of men	339 (79.2%)	85 (85.9%)	OR=6.7 (−2.5 to 14.1), P=0.13
No (%) married^*^	308 (72.0%)	81 (81.8%)	OR=9.8 (−0.2 to 18.2), P=0.05
Median (IQR) time in full time education (years)	14.0 (12 to 17.0)	14.8 (12.0 to 17.0)	W statistic=19 448,^†^ P=0.2
Mean (SD) full scale IQ (estimated from TOPF)^*^^†^	117.4 (10.6)	120.0 (8.3)	MD=−2.6 (−4.5 to −0.7), P=0.009
No (%) by social class^‡^:			
1	62 (15.5)	21 (21.2)	Pearson statistic=7.4,^§^ P=0.4
2	328 (76.6)	76 (76.8)	
3	34 (7.9)	2 (2.0)	
4	4 (0.9)	0	
No (%) of smokers^*^	11 (2.6%)	11 (11.1%)	OR=8.5 (2.7 to 16.5), P<0.001
Mean (SD) systolic blood pressure^*^	140.3 (17.8)	143.3 (16.7)	MD=−3.0 (−6.9 to 0.8), P=0.1
Mean (SD) diastolic blood pressure^*^	76.3 (10.5)	77.7 (10.9)	MD=−1.4 (−3.7 to 0.9), P=0.2
Median (IQR) Framingham stroke risk total score (10 year probability, %)^*^	9 (6.0 to 15.0)	11 (5.3 to 16.8)	OR=2.0 (−4.2 to 10.3), P=0.5
No (%) with history of major depressive disorder (%)^*^^§^	79 (18.5%)	16 (16.2%)	OR=2.3 (−7.2 to 10.1), P=0.6
Mean (SD) social visits (weekly)^*^	4.4 (3.2)	3.9 (3.1)	MD=0.5 (-0.2 to 1.2), P=0.2
No (%) taking psychotropic drugs^*^	62 (14.5%)	12 (12.1%)	OR=2.4 (−6.3 to 9.2), P=0.5
Median (IQR) MoCA total (/30)^*^	28 (26.0 to 29.0)	28 (26.0 to 29.0)	OR=0.0 (−1.7 to 4.5), P=1.0
Median (IQR) MMSE baseline total (/30)^¶^	29 (28.0 to 30.0)	29 (28.0 to 30.0)	OR=0.0 (−1.7 to 4.5), P=1.0

MD=mean difference; OR=odds ratio; MoCA=Montreal cognitive assessment.

*At time of scan.

†Test of premorbid function.

‡Social class based on occupation at phase 3: 1=professional, 2=managerial, 3=skilled non-manual, 4=skilled manual.

§Structured clinical interview for DSM IV (SCID).

¶Phase 7 (n=389).

Median alcohol consumption across study phases (fig 3 and appendix table B) was 11.5 units (85.8 g) a week (interquartile range 6.2-18.8 units (51.7-154.3 g)) for men and 6.4 units (51.4 g) a week (2.8-11.9 units (22.7-103.6 g)) for women. Weekly alcohol intake did not significantly increase over the phases of the study for the group as a whole (change in weekly alcohol units per 10 years of follow-up 0.15, 95% confidence interval −0.21 to 0.51; P=0.4), but trends over time correlated with baseline intake (intercepts and slopes correlated negatively (*r*= −0.43, 95% confidence interval −0.50 to −0.36)—that is, those drinking more at baseline tended to lower their consumption more over the course of the study, a finding consistent with regression to the mean. Male sex (difference in weekly alcohol units compared with women 4.89, 2.54 to 7.19; P<0.001) and higher premorbid IQ (change in weekly alcohol units for every 1 IQ point 0.18, 0.06 to 0.30; P=0.004) predicted higher baseline consumption but not changes in consumption with time. Other sociodemographic and clinical factors were not related to consumption. Average alcohol use over the study was over “safe limits” in 13.6% women and 20.0% men, as judged by pre-2016 UK guidelines (>21 units (168 g)/week for men, >14 units (112 g)/week for women), and 40.3% as judged by the 2016 revised UK guidelines (>14 units (112 g)/week for men and women) (see appendix for consumption data for single phases). Scores on the CAGE questionnaire were below the sensitive screening cut off of 2^28^ for all participants at all Whitehall II phases (appendix table C).

**Figure f3:**
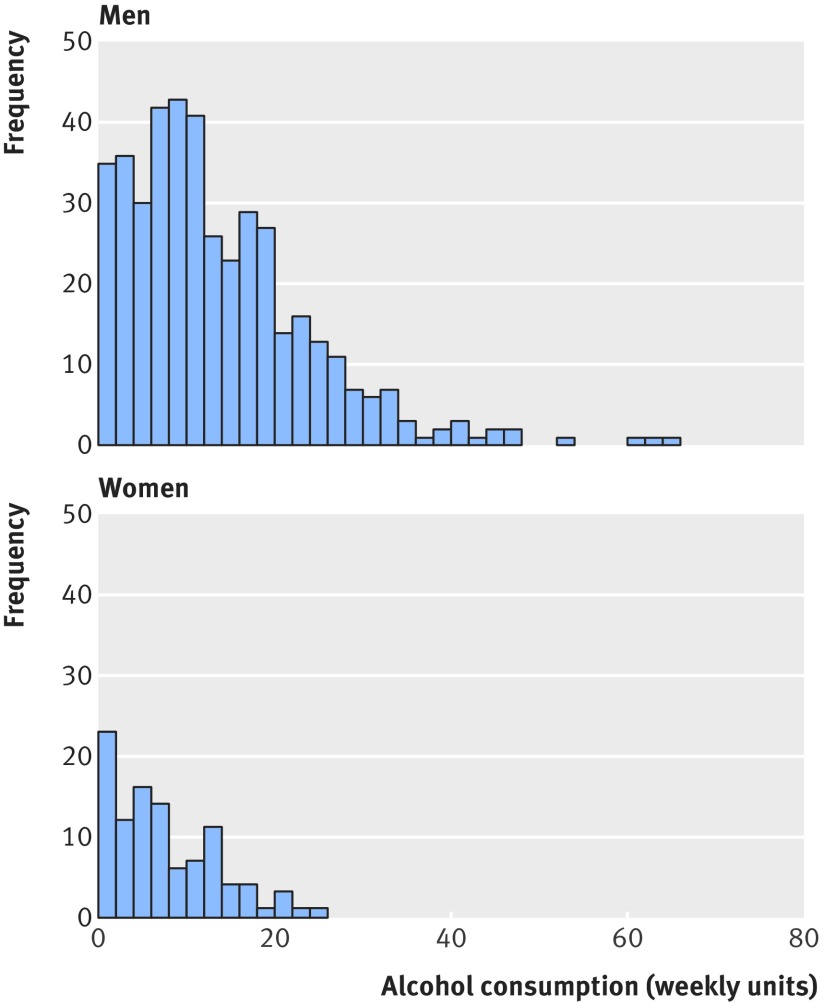
**Fig 3** Frequency distribution of alcohol consumption on average across study by sex

### Alcohol and brain structure

Higher alcohol use was associated with reduced grey matter density, hippocampal atrophy, and reduced white matter microstructural integrity.

#### Grey matter

Average alcohol consumption over the study (units/week) was negatively correlated with grey matter density in the voxel based morphometry analyses, especially in hippocampi (fig 4), even after adjustment for multiple potential confounders. Associations also extended anteriorly into the amygdalae. Frontal regions were unaffected.

**Figure f4:**
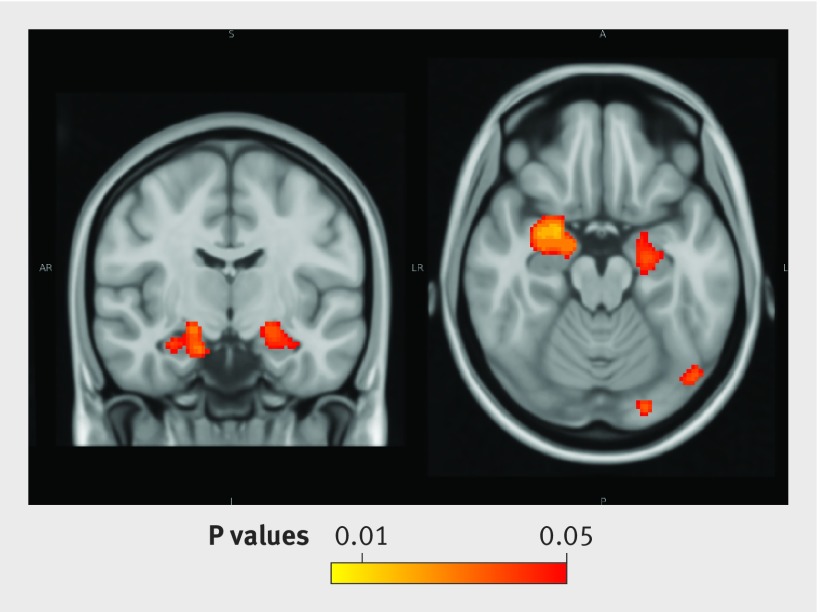
**Fig 4** Results of voxel based morphometry (corrected for threshold-free cluster enhancement (TFCE)): significant negative correlation between weekly alcohol units (average of all phases across study) and grey matter density in 527 participants. Adjusted for age, sex, education, premorbid IQ, social class, physical exercise, club attendance, social activity, Framingham stroke risk score, psychotropic drugs, and history of major depressive disorder

Compared with abstinence, higher alcohol consumption was also associated with increased odds of abnormally rated hippocampal atrophy (defined as score >0 on Scheltens visual rating scale; table 2). This was a dose dependent effect. The highest odds were in those drinking in excess of 30 units a week (odds ratio 5.8, 95% confidence interval 1.8 to 18.6; P≤0.001), but odds of atrophy were higher compared with abstinence even in those drinking at moderate levels of 7-<14 units a week (3.4, 1.4 to 8.1; P=0.007). There was no protective effect (that is, reduced odds of atrophy) with light drinking (1-<7 units a week) over abstinence. Findings were similar in subanalyses of men alone but not in the smaller subgroup of women. The risk of right sided hippocampal atrophy was significantly greater at >14 alcohol units a week compared with abstinence, but for left sided atrophy at only >30 units a week.

**Table 2 tbl2:** Adjusted^*^ odds ratios for left and right sided hippocampal atrophy on Scheltens visual rating score (reference based on abstainers), with average alcohol consumption (abstinence (<1 unit) is reference category) in 527 participants. Figures are numbers with hippocampal atrophy and total numbers in drinking category with odds ratios (95% confidence interval), and P values

Alcohol (units weekly)	Right hippocampal atrophy (*v* none)				Left hippocampal atrophy (*v* none)		
	No (total)	OR (95% CI)	P value		No (total)	OR (95% CI)	P value
Men							
0-<1	9 (22)	—	—		12 (22)	—	—
1-<7	55 (99)	1.6 (0.6 to 4.3)	0.4		69 (99)	1.7 (0.6 to 4.8)	0.3
7-<14	68 (132)	1.7 (0.6 to 4.5)	0.3		85 (132)	1.5 (0.6 to 4.2)	0.4
14-<21	57 (86)	3.2 (1.1 to 9.3)	0.02		59 (86)	2.0 (0.7 to 5.7)	0.2
21-<30	38 (54)	3.9 (1.3 to 12.0)	0.02		39 (54)	2.2 (0.7 to 6.8)	0.2
≥30	24 (31)	5.2 (1.4 to 19.0)	0.01		27 (31)	6.3 (1.5 to 27.0)	0.01
Women							
0-<1	4 (15)	—	—		7 (15)	—	—
1-<7	12 (41)	1.1 (0.2 to 5.6)	0.9		20 (41)	0.8 (0.2 to 3.6)	0.8
7-<14	19 (33)	3.1 (0.6 to 16.6)	0.2		22 (33)	2.0 (0.4 to 10.2)	0.4
14-<21	6 (11)	4.2 (0.6 to 28.8)	0.1		9 (11)	6.2 (0.7 to 55.2)	0.1
21-<30	1 (3)	1.1 (0.04 to 26.9)	1.0		2 (3)	0.4 (0.02 to 8.4)	0.4
≥30	4 (15)	—	—		7 (15)	—	—
Total							
0-<1	13 (37)	—	—		19 (37)	—	—
1-<7	67 (140)	1.5 (0.7 to 3.4)	0.3		89 (140)	1.3 (0.6 to 3.0)	0.5
7-<14	87 (165)	2.0 (0.9 to 4.4)	0.1		107 (165)	1.4 (0.6 to 3.2)	0.4
14-<21	63 (97)	3.4 (1.4 to 8.1)	0.007		68 (97)	1.9 (0.8 to 4.6)	0.1
21-<30	39 (57)	3.6 (1.4 to 9.6)	0.009		41 (57)	1.9 (0.7 to 4.9)	0.2
≥30	24 (31)	5.8 (1.8 to 18.6)	<0.001		27 (31)	5.7 (1.5 to 21.6)	0.01

*Adjusted for age, sex, premorbid IQ, education, social class, Framingham risk score, history of major depressive disorder (SCID), exercise frequency, club attendance, social visits, current use of psychotropic drugs.

Mean hippocampal volumes (raw and adjusted for intracranial volume) were within the range cited in the literature (appendix table D)^38^ ^39^ ^40^ and correlated with visual ratings of hippocampal atrophy (Spearman’s *r*=−0.4; P<0.001). Consistent with voxel based morphometry and visual ratings findings, alcohol consumption independently predicted FIRST-extracted hippocampal volume (%ICV) (table 3). Exclusion of the three individual highest drinkers (>60 units weekly) did not substantially change the results (appendix table E). In the subset of participants for whom personality trait data were available from phase 1 (n=179), additionally adjustment for the analysis for trait impulsivity did not alter the findings.

**Table 3 tbl3:** Multiple linear regression results, with squared hippocampal volume (% of intracranial volume) as dependent variable and average weekly alcohol consumption across study as independent variable

	Change in volume for every 10 unit increase in consumption (95% CI)	P value
Unadjusted alcohol	−0.26 (−0.37 to −0.15)	<0.001
Adjusted alcohol^*^	−0.19 (−0.30 to −0.08)	<0.001

*Adjusted for age, sex, premorbid IQ, education, social class, marital status, Framingham stroke risk score, history of major depressive disorder (SCID), exercise frequency, club attendance, social visits, current use of psychotropic drugs.

#### White matter

Higher average alcohol consumption across the study was inversely associated with white matter integrity (fig 5), reflected by lower corpus callosum fractional anisotropy and higher radial, axial and mean diffusivity. These associations were focused on the anterior corpus callosum (genu and anterior body, fig 5).

**Figure f5:**
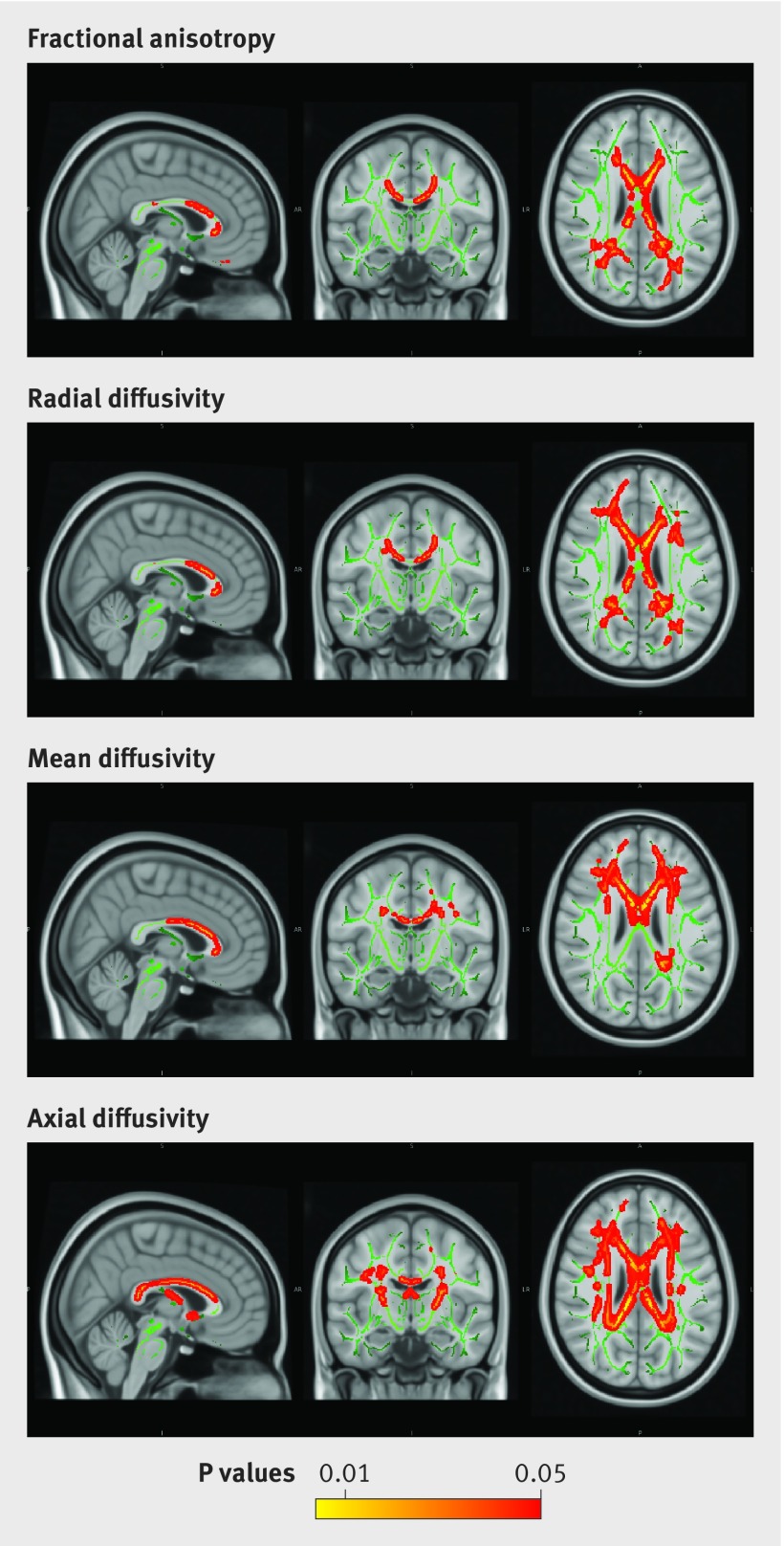
**Fig 5** Tract based spatial statistics results (corrected for threshold-free cluster enhancement, TFCE) showing negative correlation between average alcohol across study (all phases) and fractional anisotropy, and positive correlations with radial diffusivity, mean diffusivity, and axial diffusivity in 511 participants. Adjusted for age, sex, education, premorbid IQ, social class, physical exercise, club attendance, social activity, Framingham stroke risk score, psychotropic drugs, and history of major depressive disorder

### Alcohol and cognitive function

Higher alcohol consumption over the study predicted faster decline on lexical fluency but not semantic fluency or word recall (fig 6). Those drinking 7-<14, 14-<21, and >21 units a week declined faster in terms of lexical scores than abstainers. This effect was independent of age, sex, premorbid IQ, education, social class, and Framingham stroke risk score.****The size of the difference can be interpreted as follows: people drinking 7-<14 units experienced a 0.5% greater reduction from their baseline in lexical fluency per year (14% over 30 years), those drinking 14-<21 units 0.8% greater per year (17% over 30 years), and those drinking >21 units 0.6% per year (16% over 30 years) than abstainers (appendix table F). Though the three categories of higher consumption (7-<14, 14-<21, and >21 units/week) showed significantly greater decline than abstainers, the only significant difference in trends between these three groups was between those drinking 14-21 units and those drinking 7-14 units (14-21 units experience 0.3% faster decline per year; P=0.02). There was no evidence to support light drinkers being relatively protected from cognitive decline compared with abstainers. Overall results of tests examining the question of whether rates of cognitive decline are linked to alcohol were significant (after multiple comparisons correction) for lexical fluency (χ^2^=14.4; P=0.006) but not semantic fluency (χ^2^=10.0; P=0.04) or memory recall (χ^2^=9.8; P=0.04).

**Figure f6:**
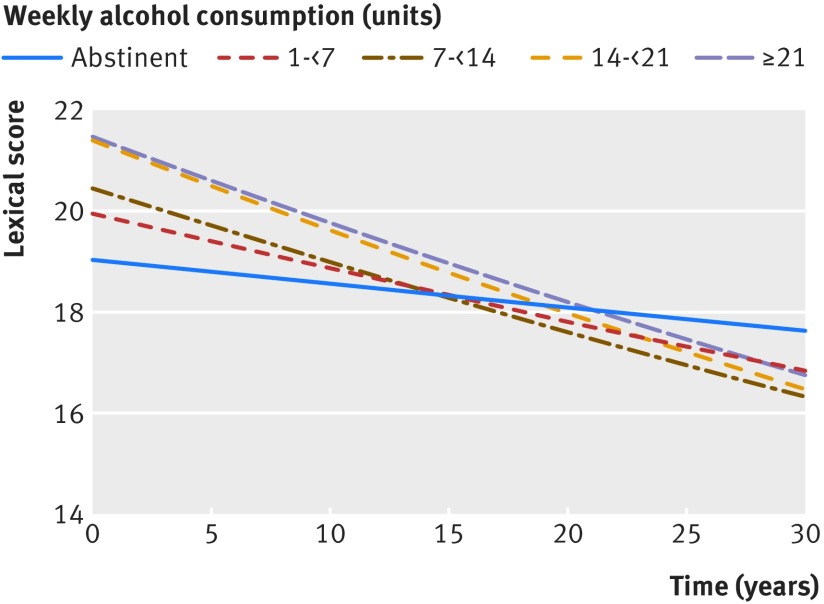
**Fig 6** Predicted longitudinal change in cognitive test scores (lexical and semantic fluency, word recall “memory”) for man of mean age (70) and premorbid IQ (118), median education (15 years), social class I and Framingham stroke risk score (10%) according to average alcohol consumption (weekly units). Predictions made on basis of mixed effects models with cognitive testing performed at phases 3, 5, 7, 9, and 11 and time of scan

We found evidence of learning effects on lexical and categorical fluency tests (P≤0.01), such that the second time a participant was presented with a test they performed better. This learning effect was predicted by premorbid IQ (First*premorbid IQ P=0.002-0.02). 

There was a trend towards higher baseline performance on lexical fluency and memory recall in those drinking compared with abstainers (appendix table F), but these findings did not reach significance after correction for multiple testing.

We did not find any significant relations between alcohol consumption and cross sectional performance on cognitive tests performed at the time of scanning (a summary of cognitive test performance and its relation to alcohol is given in appendix table H).

### Modelling alcohol consumption and brain structure and function

To see how alcohol consumption and the associated brain regions interacted with cognitive decline, we used structural equation modelling. Hippocampal volume and corpus callosum mean diffusivity were included as exogenous variables. Age, sex, and premorbid FSIQ were also incorporated.

Removal of regression arrows from age, sex, premorbid IQ, and hippocampal volume to lexical fluency decline improved the model fit. Alcohol consumption independently predicted decline in lexical fluency. The final parsimonious model explained 21% of corpus callosum mean diffusivity, 14% of right hippocampal volume, and 2% of lexical fluency decline variance (fig 7, table 4), with good model fit. Alcohol consumption (in addition to age) predicted smaller hippocampal volume and greater corpus callosum mean diffusivity. Through its relation with corpus callosum mean diffusivity, and through a direct path, increased alcohol consumption predicted faster decline of lexical fluency.

**Figure f7:**
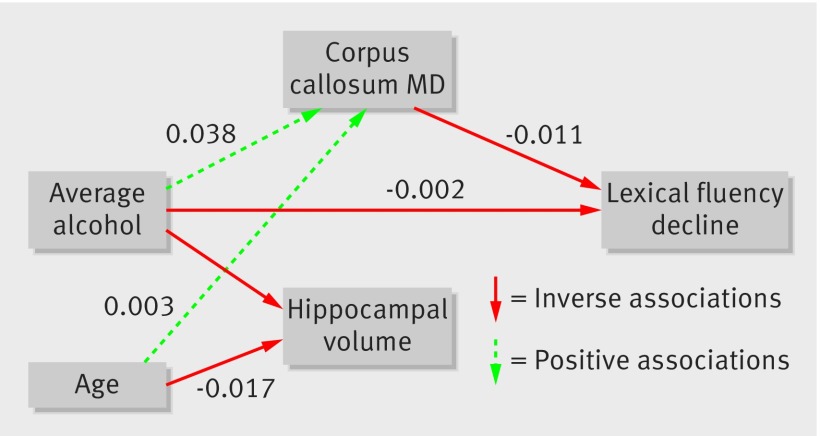
**Fig 7** Final parsimonious structural equation model illustrating relations among alcohol consumption (average across study phases, as fraction of 100 units weekly), hippocampal volume (average, %intracranial volume), corpus callosum mean diffusivity (as multiplicative of 1000), decline in lexical fluency (slopes), and age in 511 participants. Values on arrows represent unit changes in dependent variable for 1 unit increase in predictor. Model explained 21% of corpus callosum mean diffusivity, 14% of hippocampal variance, and 2% of lexical fluency decline variance (R^2^). Model fit: χ^2^=5.6, df=4, P=0.23, root mean square error of approximation=0.03, comparative fit index=0.99, Tucker-Lewis index=0.97

**Table 4 tbl4:** Parameter estimates for paths in final structural equation model (fig 6), with their bias corrected 95% confidence intervals and P values, in 511 participants

Path		Change in y for each unit increase in x (95% CI)	P value
From (x)	To (y)		
Average alcohol^*^	Hippocampal volume	−0.572 (−0.800 to −0.353)	0.01
Average alcohol	Corpus callosum mean diffusivity^†^	0.038 (0.017 to 0.064)	0.009
Average alcohol^*^	Lexical fluency decline	−0.002 (−0.005 to 0.000)	0.08
Corpus callosum mean diffusivity^†^	Lexical fluency decline	−0.011 (−0.022 to −0.003)	0.003
Age	Hippocampal volume	−0.020 (−0.021 to −0.013)	0.008
Age	Corpus callosum mean diffusivity^2^	0.003 (0.002 to 0.003)	0.03

*As fraction of 100 units weekly.

†As multiplicative of 1000.

## Discussion

### Principal findings

We have found a previously uncharacterised dose dependent association between alcohol consumption over 30 years of follow-up and hippocampal atrophy, as well as impaired white matter microstructure. Additionally, higher alcohol consumption predicted greater decline in lexical fluency but not in semantic fluency or word recall. There was no evidence of a protective effect of light drinking over abstinence on brain structure or function. The hippocampal findings were consistent between the brain-wide voxel based approach, automatically extracted volumes, and clinical visual ratings of hippocampal atrophy. The relation was dose dependent, and increased odds of hippocampal atrophy were found even in moderate drinkers (14-<21 units/week in men). The association between alcohol consumption and white matter microstructure in non-dependent drinkers is also novel and seemed to be driven by greater radial relative to axial diffusivity.

### Strengths and limitations

Strengths of this study are the 30 year longitudinal data on alcohol consumption and the detailed available data on confounders. Additional strengths include the availability of a large amount of MRI data and the advanced methods of imaging analysis. Grey matter findings were replicated with a voxel based approach, automated hippocampal volumes, and visual ratings. Visual atrophy ratings are known to correlate closely with automated methods (own data) and are more applicable to clinical settings.^41^ In large neuroimaging studies, automatic segmentation is widespread.^42^ ^43^ The automated approach we use (FIRST) has been shown to give accurate and robust results.^44^

When interpreting these results, some caveats are necessary. While the sample comprised people living in the community, it might not be representative of the wider UK population. Most participants were educated and middle class men. The hippocampal atrophy associations we found in the total sample were replicated in men alone but not in women. This could reflect a lower power to detect an effect in women, in part because the sample was dominated by men (a reflection of the sex disparity in the civil service in the 1980s) and in part because few of the included women drank heavily. This is an observational study as long term alcohol use cannot be randomised. The Rosenthal effect could have influenced participants to lead healthier lifestyles as they were enrolled in the Whitehall II “stress and health” study. Data on alcohol use were self reported, and participants could have underestimated their drinking, though the longitudinal rather than cross sectional approach often taken in other reported studies might minimise this,^27^ and the percentage of people drinking “unsafely” was comparable with that reported elsewhere.^45^ ^46^ ^47^ We used the CAGE screening instrument to identify alcohol dependence as it is well validated.^28^ ^48^ There were 75 (14.2%) individuals with missing CAGE data from at least one phase, and we cannot exclude the possibility that we have included some people who were alcohol dependent at points during the study period. All included individuals, however, had at least three (out of a total of five) CAGE measurements, and individuals with incomplete CAGE data on average drank significantly less than those with complete data (on a *t* test of means of 13.1 (SD 10.3) *v* 8.5 (SD 8.8) (P<0.001). Additionally, some participants reported drinking high levels of alcohol while screening negative on the CAGE, indicating a further possible inclusion of people with an alcohol use disorder in the sample. Increased odds of hippocampal atrophy and faster lexical fluency decline, however, were found even in those drinking moderate amounts. Although the alcohol and cognitive data were longitudinal, the analyses with MRI measures were cross sectional, raising the possibility that the associations between brain structure and alcohol were the result of a confounding variable. Longitudinal imaging over more than a couple of years adds further confounders as the physical scanner and imaging sequences are unlikely to be the same because of developments in MRI science, making results difficult to interpret. While efforts have been made to control for multiple potential sources of confounding, residual confounding from unmeasured sources is conceivable. To produce the adjusted associations we found, however, any uncontrolled confounders would need to be associated with both alcohol consumption and risk of brain abnormalities and unrelated to the multiple factors we controlled for. We cannot exclude the possibility, of unlikely face validity, that those with hippocampal atrophy at study baseline were more likely to drink more. Multiple testing and the possibility of a false positive is a concern when cognitive decline on three tests is performed. The small P values (range 0.015-0.004) for lexical decline according to differing alcohol consumption, which reach significance with a strict Bonferroni correction (that is, a reduced significance threshold of P<0.017), however, make this unlikely. In contrast, we cannot be as confident about the differences in baseline cognition for drinkers compared with abstainers (P=0.03).

Finally, we fitted a structural equation model for alcohol, brain, and cognitive data that was defined post hoc. As such, results of previous analyses affected the choice of included variables meaning that the fit of the model might be overoptimistic.

### Comparison with other studies

On average, 20% of men and 14% of women were drinking above pre-2016 UK guidelines (>21 units/>14 units/week, respectively). Other studies vary in reported rates of heavy drinking, but our rates are comparable.^45^ ^46^ Alcohol consumption might vary with country, as highlighted by a study using the WHO global alcohol database.^47^

Hippocampal atrophy is a sensitive and relatively specific marker of Alzheimer’s disease,^49^ though it has also been reported in chronic alcoholics.^19^ ^50^ The brain regions most vulnerable to alcohol abuse are said to be the frontal lobes.^21^ In our sample, higher but non-dependent alcohol use was not associated with subsequent frontal brain atrophy or impaired cognition. Only the study by Den Heijer and colleagues has reported hippocampal findings in non-dependent drinkers.^51^ This used a manual tracing rather than voxel based or visual rating approach to estimate hippocampal size. They reported a protective effect of moderate alcohol intake compared with abstinence, which conflicts with our results.^19^ Alcohol consumption, however, was determined cross sectionally, making it difficult to exclude reverse causation. In contrast, because of the longitudinal cognitive component of our study we could show an association between higher alcohol consumption and cognitive decline. Additionally, several known confounders of hippocampal size, such as depression, were not controlled for in the Den Heijer study.^51^ Other studies in non-dependent drinkers have reported either no effect^52^ ^53^ or a negative correlation with global grey matter but not hippocampal atrophy.^17^ ^18^ In contrast with our first hypothesis and the findings of some other studies,^11^ ^12^ ^19^ ^54^ we observed no evidence of a protective effect of light drinking compared with abstinence on brain structure or cognitive function. Previous studies did not control for (premorbid) IQ,^11^ ^12^ and only a few for socioeconomic class.^55^ ^56^ ^57^ The observed protective effect could be due to confounding as we and others found a positive association between alcohol intake and IQ.^58^ These factors separately predict better performance on cognitive tests. Supporting our second hypothesis, we found heavier alcohol consumption to be associated with adverse brain outcomes. The biological mechanism for this is unclear. Ethanol and acetaldehyde (a metabolite) are neurotoxic^59^ and cause reduced numbers^60^ ^61^ and morphological changes in hippocampal neurones in animal models.^62^ Associated thiamine and folate deficiency,^63^ repeated head trauma, cerebrovascular events, liver damage, and repeated intoxication and withdrawal have also been implicated in more severe drinkers. The risk of hippocampal atrophy might be stronger and at lower levels of alcohol consumption for the right side. More severe hippocampal atrophy on the right has been described in those at higher risk of Alzheimer’s disease (asymptomatic ApoE4 homozygotes),^64^ as well as in those with mild cognitive impairment or Alzheimer’s disease.^65^ We found no structural laterality in associations with cognitive function. The literature on this is scarce and conflicting. Stronger associations between right hippocampal volume and visuospatial memory have been reported.^66^

The voxel based morphometry analysis also showed associations between increased alcohol consumption and reduced grey matter density in the amygdala. This result could not be confirmed with other methods as automated segmentation of these regions was unreliable, and we are unaware of any reliable visual atrophy rating scales. Amygdala atrophy has been described in those with Alzheimer’s disease^67^ and is implicated in preclinical models of alcohol misuse,^68^ alcohol abuse relapse,^69^ and in abstinent alcoholics,^70^ though others have found no association with lower levels of consumption.^53^

In animals, radial diffusivity reflects differences in myelination.^71^ ^72^ Previous studies have highlighted the corpus callosum as an area affected in fetal alcohol syndrome^73^ and in chronic alcoholism in Marchiafava-Bignami disease.^74^ ^75^ One study reported increased mean diffusivity in the amygdala in a post hoc analysis of female non-dependent drinkers.^25^ We are not aware of any studies investigating microstructural changes in white matter in moderate drinkers using a data driven skeletonised tract approach to diffusion tensor images, such as tract based spatial statistics. Alternative voxel-wise methods could compromise optimal analysis of multiple participants as there are alignment problems causing potential difficulties with interpretation of voxel-wise statistics.^32^

Participants drinking higher levels of alcohol over the study experienced a faster decline of lexical fluency compared with abstainers. Lexical fluency involves selecting and retrieving information based on spelling (orthography) and has characteristically been associated with frontal executive function,^76^ in contrast with semantic fluency, which could depend more on temporal lobe integrity.^77^ The distinction might not be as clear cut, however, as functional networks overlap.^78^ The inverse relation between alcohol consumption and lexical decline was perhaps unsurprising given the frontal predominance of the negative associations with white matter integrity. We suggest two possibilities for the lack of more widespread associations with cognition, particularly with semantic fluency and short term memory decline, given the structural brain findings (hippocampal atrophy). Firstly, there are clear practice effects over the study—that is, at least some participants improve their performance after repeated testing, and this is positively associated with premorbid IQ. This might be greater for the semantic compared with lexical fluency tests. Variables predicting the ability to learn could be different from those protecting against cognitive impairment because of a neurodegenerative process. Though we attempted to control for both IQ and learning effects, this might be insufficient to remove the confounding effect if a third variable, such as diet, mediates the relation between IQ and learning but is not in the model. Secondly, the brain changes might reflect an intermediate phenotype, and cognitive change is not yet evident. It is now well documented that hippocampal atrophy precedes symptoms in those with Alzheimer’s dementia by several years,^79^ so a similar phenomenon in alcohol related changes is plausible.

### Conclusions and policy implications

Prospective studies of the effects of alcohol use on the brain are few, and replication of these findings in other populations will be important. Alcohol consumption for individuals was remarkably stable across the study phases. This sample was therefore underpowered to detect differences in those considerably changing their intake from others who drink consistently. Investigations with larger numbers are needed to clarify whether there are graded risks between short versus long periods of higher alcohol consumption.

The finding that alcohol consumption in moderate quantities is associated with multiple markers of abnormal brain structure and cognitive function has important potential public health implications for a large sector of the population. For example, in our sample nearly half of the men and a quarter of the women were currently drinking in this range. Additionally, drinking habits were remarkably stable over a 30 year period, suggesting that risky drinking habits might be embarked on in midlife. Recommended guidelines for drinking remained unchanged in the UK from 1987 until 2016. Our findings support the recent reduction in UK safe limits and call into question the current US guidelines, which suggest that up to 24.5 units a week is safe for men, as we found increased odds of hippocampal atrophy at just 14-21 units a week, and we found no support for a protective effect of light consumption on brain structure. Alcohol might represent a modifiable risk factor for cognitive impairment, and primary prevention interventions targeted to later life could be too late.

**What is already known on this topic**

Heavy drinking is associated with Korsakoff’s syndrome, dementia, and widespread brain atrophy
While smaller amounts of alcohol have been linked to protection against cognitive impairment, few studies have examined the effects of moderate alcohol on the brain
Previous studies have methodological limitations especially regarding the lack of prospective alcohol data, have been conflicting, and have failed to provide a convincing neural correlate

**What this study adds**

Compared with abstinence, moderate alcohol intake is associated with increased risk of adverse brain outcomes and steeper cognitive decline in lexical fluency
The hippocampus is particularly vulnerable, which has not been previously linked negatively with moderate alcohol use
No protective effect was found for small amounts of alcohol over abstinence, and previous reports claiming a protective effect of light drinking might have been subject to confounding by associations between increased alcohol and higher social class or IQ

## Web Extra

Extra material supplied by the author

Appendix: Supplementary materials: methods and results

## Notes

We thank the Whitehall II cohort participants for their time and D Lunn for statistical advice to the corresponding author.

Ethical approval: This study was approved as part of a larger study (Predicting MRI abnormalities with longitudinal data of the Whitehall II sub- study; MSD/IDREC/C1/2011/71) by the University of Oxford medical sciences interdivisional research ethics committee.

Data sharing: Policy referenced on: https://www.psych.ox.ac.uk/research/neurobiology-of-ageing/research-projects-1/whitehall-oxford. Data will be shared a period of two years after collection to allow the research group and collaborators time for analysis and publication. Reference to Whitehall II Data sharing policy here: http://www.ucl.ac.uk/whitehallII/data-sharing.

Transparency: The lead authors affirm that the manuscript is an honest, accurate, and transparent account of the study being reported; that no important aspects of the study have been omitted; and that any discrepancies from the study as planned (and, if relevant, registered) have been explained.
