# Supplementary material for: Moderate alcohol consumption as risk factor for adverse brain outcomes and cognitive decline: longitudinal cohort study
Source: BMJ. 2017 Jun 6;357:j2353. doi: 10.1136/bmj.j2353 (PMC5460586; doi:10.1136/bmj.j2353)
Supplement: Supplementary file 1 — Appendix: Supplementary materials: methods and results [file topa034088.ww1.pdf]

Appendix: Technical details, supplementary tables, and supplementary figures [posted as supplied by author]

MRI analysis

Tissue segmentation

T1-weighted images were processed using FSL tools (FMRIB Software Library, www.fmrib.ox.ac.uk/fsl) and 'fsl_anat (Beta version)' (http://fsl.fmrib.ox.ac.uk/fsl/fslwiki/fsl_anat). This reorients images to standard (MNI) space, corrects for bias field, registers the images to standard space (using linear FLIRT(1, 2) and non-linear FNIRT(3) registration), and extracts whole brain volumes ('BET').(4) FMRIB's Automated Segmentation Tool (FAST) allowed extraction of measures of total grey matter (GM), white matter (WM) and cerebrospinal fluid (CSF). GM and WM volumes were adjusted for total intracranial volume.

Voxel-based morphometry (VBM)

Relationships between alcohol use and grey matter were examined initially using voxel-based morphometry, an objective method to compare grey matter density between individuals in each voxel (smallest distinguishable image volume) of the structural image. Structural data were analysed with FSL-VBM.(5) http://fsl.fmrib.ox.ac.uk/fsl/fslwiki/FSLVBM), an optimised VBM protocol(6) carried out with FSL tools.(7) First, structural images were brain-extracted and grey matter-segmented before being registered to the MNI 152 standard space using non-linear registration.(3) The resulting images were averaged and flipped along the x-axis to create a left-right symmetric, study-specific grey matter template. Second, all native grey matter images were non-linearly registered to this study-specific template and "modulated" to correct for local expansion (or contraction) due to the non-linear component of the spatial transformation. The modulated grey matter images were then smoothed with an isotropic Gaussian kernel with a sigma of 3 mm. Finally, voxelwise, a Generalised Linear Model (GLM) was applied using permutation-

based non-parametric testing, correcting for multiple comparisons across space (threshold-free cluster enhancement, tfce).

Visual rating of hippocampal atrophy

Structural T1 scans were assessed independently, by three researchers for hippocampal atrophy (HA) using the Scheltens scale according to the width of the choroid fissure, width of the temporal horn, and height of the hippocampus (0-4).[8] Raters remained blind to all other participant data. Intra- (on a random 10% of 208 scans) and inter-rater reliability (n=208) for visual rating scores was high (intra-class correlation coefficients: 0.8 to 0.9 and 0.7 to 0.9, respectively). Left and right hippocampal atrophy was defined independently according to visual rating (Scheltens score(9) >0) by three clinicians, who reached a consensus.

Hippocampal volumes

Hippocampal volumes were calculated using FIRST (http://fsl.fmrib.ox.ac. uk/fsl/fslwiki/FIRST)[10] an automated model-based segmentation/registration tool in a two-stage process – first using all subcortical masks, and second a hippocampal mask only. Both were visually inspected to check optimal segmentation. Extracted hippocampal volumes were corrected for intracranial volume and averaged across left and right sides.

Diffusion tensor imaging

Diffusion tensor imaging (DTI) measures the directional preference of water diffusion in neural tissue and allows inferences about the structural integrity of white matter tracts. In healthy myelinated fibres diffusion is restricted perpendicular to the longitudinal axis of the fibre, i.e. it is anisotropic. Voxelwise statistical analysis of fractional anisotropy (FA), axial diffusivity (AD), radial diffusivity (RD) and mean diffusivity (MD) data was carried out using Tract-Based Spatial Statistics (TBSS).[11] This involves non-linear registration followed

by projection onto an alignment-invariant tract representation (the “mean FA skeleton”). This avoids alignment problems for multiple subjects and avoids arbitrariness of spatial smoothing extent, improving the sensitivity, objectivity and interpretability of analysis of multi-subject diffusion imaging studies.(12) Multiple diffusion indices were analysed to allow a richer investigation of localised connectivity related changes. AD describes diffusion parallel to, and RD perpendicular to the to the principal fibre direction. MD is the apparent diffusion coefficient averaged over all directions. FA reflects AD in relation to RD and is widely used as a marker of tract integrity.(13),(14) Diffusion images were corrected for head movement and eddy currents (eddy_correct) and brain masks generated using BET. Fractional anisotropy, mean diffusivity, axial diffusivity and radial diffusivity maps were generated using DTIFit (http://fsl.fmrib.ox.ac.uk/fsl/fdt) that fits a diffusion tensor model at each voxel. Tract-based spatial statistics (TBSS) were used in a 4-stage process. Pre-processing prepared images for registration to standard space. Mean fractional anisotropy (FA), diffusivity (MD), radial diffusivity (RD), axial diffusivity (AD) and skeletonized FA, MD, RD and AD images were created, and thresholded. Lastly each FA, MD, RD and AD image was projected onto the relevant skeleton and ‘randomize’ used for statistical analyses. Masks of specific white matter tracts were created using ICBM-DTI-81 white-matter labels atlas(15) and used to extract mean white matter integrity indices.

Supplementary results

Variable	MRI Sample N=527			Phase 11 Participants N=6306			Difference in means or proportions (95% CI), p values
	N	Mean/ %	S.D.	N	Mean/%	S.D.	
**Age** [years]	527	69.6	5.3	6306	69.8	5.9	-0.2 (-0.7 to 0.3), p=0.5
**Sex**	527			6306			-0.1 (-0.2 to -0.1), p<0.001
Female	103	19.5%		1947	29.3%		
Male	424	80.5%		4459	70.7%		
**Full time education** [years]	527	14.6	3.2	5101	15.1	4.2	-0.5 (-0.9 to -0.1), p=0.008
**CES-D**	527	5.0	5.8	5855	7.3	7.6	-2.3 (-3.0 to -1.6), p<0.001
**Systolic BP** [mmHg]	527	140.8	17.6	5652	127.8	16.5	13.0 (11.5 to 14.5), p<0.001
**Diastolic BP** [mmHg]	527	76.5	10.6	5652	70.8	9.9	5.7 (4.8 to 6.6), p<0.001

Table A: Comparison of the imaging sample with the Whitehall II cohort at Phase 11.

	Men								Women					
	Weekly alcohol consumption Units (grams)								Weekly alcohol consumption Units (grams)					
Phase	Age (Mean, S.D.)	N	Mean	Standard deviation	Median	Interquartile range	N (%) 'Unsafe' drinkers (Pre-2016 guidelines)	N (%) 'Unsafe' drinkers (Post-2016 guidelines)	N	Mean	Standard deviation	Median	Interquartile range	N (%) 'Unsafe' (Pre- = Post-2016 guidelines)
**1**	43.0 (5.4)	421	13.1 (104.4)	13.1 (104.6)	10.0 (80.0)	4.0 to 18.0 (32.0 to 144.0)	72 (17.1)	135 (32.1)	101	7.3 (58.4)	8.0 (64.4)	5.0 (40.0)	2.0 to 10.0 (16.0 to 80.0)	12(11.9)
**3**	48.2 (5.4)	386	12.7 (101.5)	12.0 (95.7)	10.0 (80.0)	5.0 to 18.0 (40.0 to 144.0)	78 (18.4)	124 (32.1)	90	7.6 (60.6)	8.3 (66.7)	5.0 (40.0)	1.8 to 12.0 (14.0 to 96.0)	13(14.4)
**5**	53.1 (8.8)	397	16.4 (130.8)	15.2 (121.3)	12.0 (96.0)	6.0 to 23.0 (48.0 to 184.0)	113 (28.5)	177 (44.6)	99	9.3 (74.5)	9.7 (77.4)	6.5 (52.0)	2.0 to 12.0 (16.0 to 96.0)	17(17.2)
**7**	59.5 (5.3)	410	14.3 (114.8)	12.2 (97.6)	12.0 (96.0)	5.0 to 20.0 (40.0 to 160.0)	90 (22.0)	164 (40.0)	97	8.1 (64.7)	7.5 (59.6)	6.0 (48.0)	2.0 to 14.0 (16.0 to 112.0)	16(16.5)
**9**	64.4 (5.3)	412	12.6 (101.1)	11.0 (88.6)	10.0 (80.0)	4.0 to 20.0 (32.0 to 160.0)	76 (18.4)	140 (34.0)	101	7.2 (57.5)	7.3 (58.1)	6.0 (48.0)	1.0 to 10.3 (8.0 to 82.0)	14(13.7)
**11**	68.5 (5.4)	424	11.7 (94.3)	10.6 (85.5)	10.0 (80.0)	4.0 to 17.0 (32.0 to 136.0)	62 (14.6)	121 (28.5)	103	5.6 (45.1)	5.7 (45.2)	4.0 (32.0)	0.8 to 9.0 (6.0 to 72.0)	8(7.8)
**Time of Scan**	69.6 (5.3)	418	16.8 (112.3)	15.9 (122.9)	13.8 (101.7)	4.4 to 21.9 (34.8 to 175.6)	113 (26.7)	206 (48.6)	100	9.6 (77.1)	10.5 (84.2)	6.6 (52.8)	1.5 to 15.1 (12.0 to 120.8)	9(9.0)
**Average**		424	14.0 (111.8)	10.7 (85.8)	11.5 (92.3)	6.2 to 18.8 (51.7 to 154.3)	85 (20.0)	171 (40.3)	103	7.8 (62.7)	6.2 (49.6)	6.4 (51.4)	2.8 to 11.9 (22.7 to 103.6)	14(13.6)

Table B: Summary of alcohol consumption by gender at each study phase (n=527) including: weekly units (grams) consumed; percent drinking over safe weekly limits, as defined by pre-2016 (>14 units/112g women, >21 units/168g men), and post-2016 guidelines (>14 units/112g)

		CAGE score n (valid %)		
**Phase**	**Total with CAGE completed**	**0**	**1**	**2/3**
**3**	525	468 (88.6)	57 (11.4)	0 (0)
**5**	494	439 (89.2)	55 (10.8)	0 (0)
**7**	527	470 (88.7)	57 (11.3)	0 (0)
**9**	499	444 (88.5)	55 (11.5)	0 (0)
**11**	509	453 (88.8)	56 (11.2)	0 (0)

Table C: Summary of CAGE (screen for alcohol dependence) scores across study phases.

	Mean (S.D.)
Right hippocampal volume (unadjusted, mm^3)	3474 (433)
Left hippocampal volume (unadjusted, mm^3)	3368 (444)
Right hippocampal volume (adjusted as % of ICV)	2.42 (0.30)
Left hippocampal volume (adjusted as % of ICV)	2.35 (0.32)

Table D: Mean (S.D.) raw and adjusted hippocampal volumes as percentage of intracranial volume (%ICV) extracted using FIRST for the sample.

	Change in hippocampal volume[2] (% intracranial volume) for every 10 unit increase in weekly alcohol consumption	95% CI	P value
**Alcohol adjusted[1] (all cases)**	-0.19	-0.30 to -0.08	<0.001
**Alcohol adjusted[1] (excluding highest 3 drinkers)**	-0.18	-0.29 to -0.06	0.003

[*] For every 10 unit increase in alcohol weekly
[1] Analyses adjusted for age, sex, premorbid IQ, education, social class, marital status, Framingham Risk Score, smoking, history of Major Depressive Disorder (SCID), exercise frequency, club attendance, social visits, current psychotropic medication.
[2]Hippocampal volume squared.

Table E: Outlier analysis of multiple linear regression results, with squared hippocampal volume (% of ICV) as the dependent variable and average alcohol consumption as an independent variable.

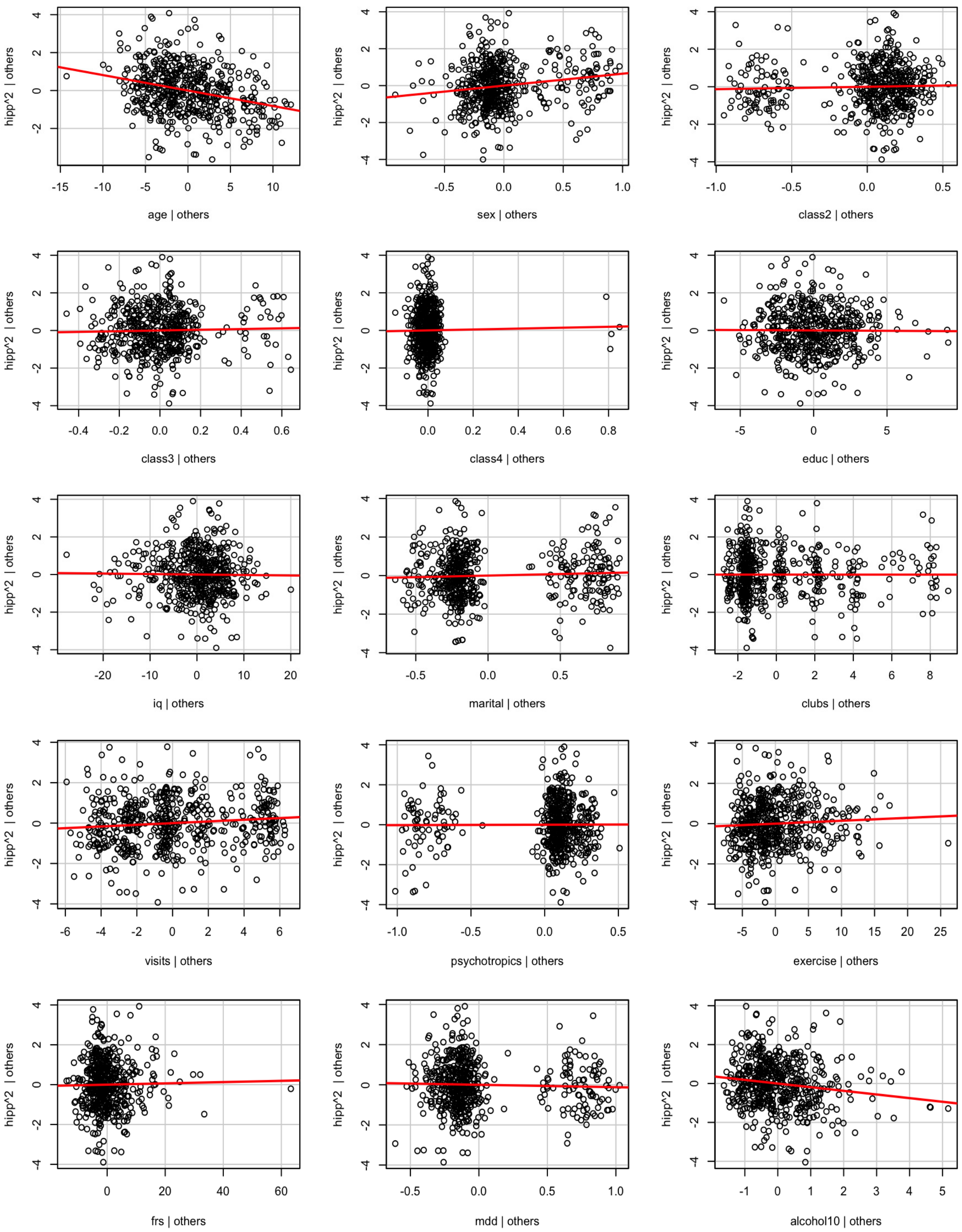

Figure A: Partial regression plots showing the relationships between a number of variables and hippocampal volume (extracted from FIRST, as %ICV, squared), controlling for other variables in the model. For example, the last plot visualises the relationship between alcohol and hippocampal size, after controlling for: age, sex, social class, education, FSIQ, marital status, club participation, social visits, psychotropic medication, exercise, Framingham Risk Score and history of Major Depressive Disorder. The x-axis units are residuals from a regression model omitting alcohol. The y-axis units are residuals from a regression of alcohol against the remainder of the independent variables. The slope of the red line represents the partial regression coefficients.

	Estimates[3]	S.E.	95% CI	P value
		**LEXICAL FLUENCY**		
**Age**	0.992	0.18	0.988 to 0.995	<0.001***
**Time**	0.997	0.19	0.994 to 1.164	0.18
**Sex[1]**	1.064	0.02	1.019 to 1.111	0.005
**First**	1.422	0.14	1.073 to 1.885	0.01**
**Social class[2]**	0.937	0.02	0.895 to 0.981	0.006**
**FSIQ**	1.009	0.12	1.007 to 1.011	<0.001***
**Education**	0.995	0.04	0.988 to 1.002	0.14
**FRS**	0.998	0.12	0.996 to 1.001	0.12
**1 - <7**	1.048	0.05	0.944 to 1.164	0.38
**7 - <14**	1.075	0.05	0.969 to 1.191	0.17
**14 - <21**	1.125	0.06	1.009 to 1.254	0.03*
**>21**	1.127	0.06	1.009 to 1.259	0.03*
**Timex 1 - <7**	0.997	0.21	0.993 to 1.001	0.13
**Timex 7 - <14**	0.995	0.20	0.991 to 0.999	0.015*
**Timex 14 - <21**	0.994	0.21	0.990 to 0.998	0.004**
**Timex >21**	0.994	0.22	0.990 to 0.999	0.009**
**FSIQ x First**	0.997	0.12	0.994 to 0.999	0.005**
		**SEMANTIC FLUENCY**		
**Age**	0.989	0.16	0.986 to 0.993	<0.001***
**Time**	0.996	0.21	0.992 to 1.000	0.05
**Sex[1]**	1.065	0.02	1.024 to 1.107	0.002**
**First**	1.588	0.16	1.158 to 2.178	0.004**
**Oxford**	2.104	0.10	1.746 to 2.535	<0.001***
**Social class[2]**	0.926	0.02	0.888 to 0.965	<0.001***
**FSIQ**	1.012	0. 11	1.010 to 1.014	<0.001***
**Education**	0.990	0.03	0.984 to 0.997	0.03*
**FRS**	0.999	0.11	0.996 to 1.001	0.15
**1 - <7**	1.000	0.05	0.899 to 1.112	1.0
**7 - <14**	1.060	0.05	0.954 to 1.178	0.28
**14 - <21**	1.068	0.06	0.956 to 1.193	0.25
**>21**	1.090	0.06	0.973 to 1.221	0.14
**Time x 1 - <7**	1.001	0.22	0.997 to 1.006	0.58
**Time x 7 - <14**	0.998	0.22	0.994 to 1.003	0.47
**Time x 14 - <21**	0.999	0.23	0.994 to 1.003	0.50
**Time x >21**	0.998	0.23	0.994 to 1.003	0.41
**FSIQ x First**	0.996	0.13	0.993 to 0.998	0.002**
**FSIQ x Oxford**	0.997	0.08	0.995 to 0.998	<0.001***
		**WORD RECALL**		
**Age**	0.978	0.34	0.972 to 0.985	<0.001***
**Time**	0.992	0.52	0.982 to 1.002	0.13
**Sex[1]**	1.218	0.04	1.123 to 1.320	<0.001***
**First**	1.499	0.36	0.741 to 3.030	0.26
**Social class[2]**	0.925	0.04	0.848 to 1.009	0.08
**FSIQ**	1.016	0.23	1.012 to 1.021	<0.001***
**Education**	0.978	0.66	0.965 to 0.991	0.008**
**FRS**	0.996	0.22	0.992 to 1.000	0.06
**1 - <7**	1.223	0.12	0.971 to 1.566	0.09
**7 - <14**	1.299	0.12	1.026 to 1.644	0.03*
**14 - <21**	1.181	0.13	0.922 to 1.514	0.19
**>21**	1.361	0.13	1.056 to 1.753	0.02*
**Time x 1- < 7**	0.993	0.57	0.982 to 1.004	0.24
**Time x 7- < 14**	0.992	0.56	0.981 to 1.003	0.18
**Time x 14 - <21**	0.997	0.59	0.986 to 1.009	0.65
**Time x >21**	0.989	0.60	0.978 to 1.001	0.07
**FSIQ x First**	0.993	0.30	0.988 to 0.999	0.02*

*** P value <0.05**
**** P value <0.01**
***** P value <0.001**

[1] Females vs. reference group males.
[2]Classes 2,3 and 4 vs. reference group class 1.
[3]Estimates represent odds for memory recall (binomial regression) and mean count for lexical and semantic fluency (Poisson regression).

Table F: Results from mixed effects models fitting longitudinal cognitive test scores over time according to average alcohol consumption (reference group abstainers) across the study. For lexical and semantic fluency Poisson regression is applied, and for memory recall binomial regression. Adjusted for: age, sex, education, social class, FSIQ, Framingham Risk Score, learning effects of the test and time from study baseline.

Test	Change in lexical fluency slope* for every 10 unit increase in weekly alcohol consumption	95% CI	P value
Average alcohol[1]	-0.28	-0.53 to -0.02	0.03
Average alcohol[2]	-0.23	-0.46 to 0.57	0.08

[1] Adjusted age, sex, education, FSIQ
[2] As in [1] and additionally corpus callosum MD
*As a multiple of 100

Table G: Results from multiple linear regression analysis, with decline in lexical fluency (slopes, Phase 3 to time of scan) as the dependent variable, and average alcohol consumption (weekly units) as an independent variable.

Cognitive test	Mean (S.D.)[1]/ median (IQR)[2]	P value for likelihood test[3]
MoCA (adjusted for education)	28.0 (26.0 to 29.0)[2]	0.4
TMT A (seconds)	31.3 (13.4)[1]	0.7
TMT B (seconds)	68.7 (36.9)[1]	0.3
Rey copy	32.0* (29.0 to 34.0)[2]	0.6
Rey immediate	16.0 (10.8 to 20.5)[2]	0.9
Rey delay	15.5 (11.0 to 19.5)[2]	0.8
HVLT total recall	28.0 (25.0 to 31.0)[2]	0.4
HVLT delayed recall	10.0 (8.0 to 11.0)[2]	0.9
BNT	59.0 (57.0 to 60.0)[2]	0.01*
Digit span total	30.0 (27.0 to 35.0)[2]	0.8
Digit substitution test	63.0 (54.0 t0 71.0)[2]	0.3

[1] Mean (S.D.) for normally distributed continuous variables.

[2] Median (IQR) for discrete variables.

[3] Hypothesis test for significant difference between models with and without alcohol included.
* Not significant after Bonferroni correction (0.05/9 = 0.006 significance level).

Table H: Summary of cross-sectional cognitive test data and its relationship with average alcohol consumption across the study. Means and standard deviations are given for normally distributed data and medians and interquartile ranges for non-normally distributed data. MoCA=Montreal Cognitive Assessment, TMT=Trail making test, Rey=complex figure task, HVLT=Hopkins Verbal Learning Test, BNT=Boston naming test (associated with IQ, hence positive association). P values result from hypothesis tests (likelihood tests) comparing regression models with cognitive test as the dependent variable, adjusted for age, sex, education and FSIQ, with and without alcohol consumption in the model.

1. Jenkinson M, Smith S. A global optimisation method for robust affine registration of brain images. Medical image analysis. 2001;5(2):143-56.
2. Jenkinson M, Bannister P, Brady M, Smith S. Improved optimization for the robust and accurate linear registration and motion correction of brain images. Neuroimage. 2002;17(2):825-41.
3. Andersson JL, Jenkinson M, Smith S. Non-linear registration, aka Spatial normalisation FMRIB technical report TR07JA2. FMRIB Analysis Group of the University of Oxford. 2007.
4. Smith SM. Fast robust automated brain extraction. Human brain mapping. 2002;17(3):143-55.
5. Douaud G, Smith S, Jenkinson M, Behrens T, Johansen-Berg H, Vickers J, et al. Anatomically related grey and white matter abnormalities in adolescent-onset schizophrenia. Brain. 2007;130(9):2375-86.
6. Good CD, Johnsrude I, Ashburner J, Henson RN, Friston KJ, Frackowiak RS. Cerebral asymmetry and the effects of sex and handedness on brain structure: a voxel-based morphometric analysis of 465 normal adult human brains. Neuroimage. 2001;14(3):685-700.
7. Smith SM, Jenkinson M, Woolrich MW, Beckmann CF, Behrens TE, Johansen-Berg H, et al. Advances in functional and structural MR image analysis and implementation as FSL. Neuroimage. 2004;23:S208-S19.
8. Scheltens P, Leys D, Barkhof F, Huglo D, Weinstein H, Vermersch P, et al. Atrophy of medial temporal lobes on MRI in" probable" Alzheimer's disease and normal ageing: diagnostic value and neuropsychological correlates. Journal of neurology, neurosurgery, and psychiatry. 1992;55(10):967-72.
9. Scheltens P, Leys D, Barkhof F, Huglo D, Weinstein H, Vermersch P, et al. Atrophy of medial temporal lobes on MRI in" probable" Alzheimer's disease and normal ageing: diagnostic value and neuropsychological correlates. Journal of Neurology, Neurosurgery & Psychiatry. 1992;55(10):967-72.
10. Patenaude B, Smith SM, Kennedy DN, Jenkinson M. A Bayesian model of shape and appearance for subcortical brain segmentation. Neuroimage. 2011;56(3):907-22.
11. Smith SM, Jenkinson M, Johansen-Berg H, Rueckert D, Nichols TE, Mackay CE, et al. Tract-based spatial statistics: voxelwise analysis of multi-subject diffusion data. Neuroimage. 2006;31(4):1487-505.
12. Smith SM, Jenkinson M, Johansen-Berg H, Rueckert D, Nichols TE, Mackay CE, et al. Tract-based spatial statistics: voxelwise analysis of multi-subject diffusion data. Neuroimage. 2006;31(4):1487-505.
13. Johansen-Berg H, Behrens TE. Diffusion MRI: from quantitative measurement to in vivo neuroanatomy: Academic Press; 2013.
14. Koerte IK, Ertl-Wagner B, Reiser M, Zafonte R, Shenton ME. White matter integrity in the brains of professional soccer players without a symptomatic concussion. JAMA. 2012;308(18):1859-61.
15. Crain B, Mori S. MRI atlas of human white matter. Amsterdam, Elsevier; 2005.
